# Evaluation of the Psychological Status, Infertility-Associated Factors, and Erectile Function in Patients with Timely Ovulatory Intercourse Failure in China: Evidence from a Cross-Sectional Study

**DOI:** 10.1155/2020/2850507

**Published:** 2020-09-19

**Authors:** Jian Xiong, Zhonglin Cai, Chengquan Ma, Bin Yang, Jianzhong Zhang, Hongjun Li

**Affiliations:** ^1^Department of Urology, Peking Union Medical College Hospital, Peking Union Medical College, Chinese Academy of Medical Sciences, Beijing, China; ^2^Department of Urology, Affiliated Hospital of the Qingdao University, Qingdao, China; ^3^Department of Urology, Beijing Friendship Hospital, Capital Medical University, China

## Abstract

**Results:**

A total of 1128 patients were enrolled, and 264 of them (23.40%) suffered from TOIF. TOIF was positively associated with smoking, drinking, hypertension, diabetes, anxiety, unknown cause of infertility, lower semen concentration, lower frequency of intercourse, and decreased erectile function. The total IIEF-15 scores, erectile function, orgasmic function, sexual desire, intercourse satisfaction, and overall satisfaction were significantly decreased in patients with TOIF.

**Conclusion:**

TOIF is associated with lower semen concentration, anxiety, and other comorbidities such as hypertension and diabetes. Clinicians are required to focus on these associated factors in addition to improve the erectile function.

## 1. Introduction

Timely ovulatory intercourse failure (TOIF), which occurs when the males failed to penetrate their wives' vagina during the ovulatory time, may affect physical and psychosocial health and may have a significant influence on the quality of life of the patients and their partners [1]. TOIF is not a rare disorder in males of infertile couples, and epidemiological data have shown a high prevalence of TOIF in males of infertile couples in China. In 2018, Yang et al. first put forward the concept of TOIF and reported that 26.2% of the males using timely ovulatory intercourse suffered from TOIF [1]. However, although the prevalence of TOIF is high, there were only few studies that have focused on this issue and there were no clear definitions. Based on the previous study by Yang et al., a patient diagnosed with TOIF should fulfill the following criteria: (1) were aware of their partners' ovulatory time, (2) had tried sexual intercourse during their partners' ovulatory time, and (3) experienced at least three failed attempts. In the current study, we have utilized the same criteria.

Knowledge about the associated factors of TOIF is limited. A previous study has reported an association between TOIF and erectile dysfunction (ED) by using the 5-item version of the International Index of Erectile Function (IIEF-5) questionnaire. The prevalence of TOIF in males with ED (23.3%) was significantly higher than that in those without ED (8.6%). Notably, TOIF did not solely result from ED considering its nature that is firmly associated with the partners' ovulatory time. Under the traditional Chinese culture, men take the responsibility to add a generation to carry on the family line. As infertility goes on, failures of timely ovulatory sexual intercourse exacerbate the psychological distress substantially [2–4]. The prevalence of psychological disorders is high in infertile males of Chinese couples. A recent study reported that the prevalence of depression, anxiety, and a combination of both psychological symptoms was 20.8%, 7.8%, and 15.4%, respectively, in 771 infertile Chinese males [5]. Psychological stress from the traditional beliefs in China can be an important cause of TOIF. In addition, other infertility-associated factors such as duration of infertility, cause for infertility, and semen analysis results should not be ignored since these factors are directly associated with the psychological stress of these patients [6, 7].

The International Index of Erectile Function 15 (IIEF-15) questionnaire contains five parts: erectile function (IIEF-EF), orgasmic function, sexual desire, intercourse satisfaction, and overall satisfaction domain [8]. In addition to the erectile function, the orgasmic function, the sexual desire, and the patients' subjective satisfaction of their sexual intercourse can be recorded. Considering the prevalence of the psychological distress in males of infertile couples, the subjective feelings including the satisfaction and sexual desire and orgasmic function of the patients should be focused on.

In the current study, the IIEF-15 questionnaire was used to investigate the sexual function in males with or without TOIF so as to evaluate the subjective attitudes towards their sexual intercourse. In addition, data concerning the clinical features of the infertile patients, the psychological status, and the semen analysis results were collected to explore the potential associated factors of TOIF.

## 2. Materials and Methods

### 2.1. Study Design and Participants

This cross-sectional and clinic-based study focused on the male partners of the infertile couples who failed to conceive after 1 year or more of regular unprotected sexual intercourse. The participating patients were continuously enrolled from the urologic clinic of Peking Union Medical College Hospital from June 1, 2018, through December 1, 2018. The inclusion criteria were (1) men living day to day with their wives or common-law partners and planning to have a baby, (2) females failing to achieve a clinical pregnancy after engaging in regular unprotected sexual intercourse for at least 1 year, (3) men attending the andrologic clinics for infertility with at least two semen samples analyzed, and (4) men who were aware of the ovulatory time of their female partners and using timely ovulatory intercourse. The patients were excluded when they were (1) males with severe cardiovascular diseases, mental illness such as dementia and delirium, or visible genital malformation and (2) men whose wives had abnormality of the reproductive system based on gynecologic examination. The males of infertile couples would be diagnosed with TOIF if they (1) were aware of their partners' ovulatory time, (2) had tried sexual intercourse during their partners' ovulatory time, and (3) experienced at least three failed attempts. The study was completely anonymous and voluntary, and informed consent forms were obtained from all the patients. This study was approved by the Research Ethics Committee in Peking Union Medical College Hospital (ethical number: JS-1176).

### 2.2. Questionnaire Survey and Semen Analyses

A self-designed questionnaire based on the previous study [1] was used to collect the basic information of the enrolled patients including age, height, weight, residence (urban or rural), monthly income, education level, occupation (office work, labor work, or others), smoking, alcohol drinking, marital status (primary or secondary), prevalence of coexisting diseases (hypertension, diabetes, varicocele, and chronic prostatitis), type of infertility (primary or secondary), duration of infertility, cause of infertility (known or unknown), frequency of sexual intercourse, and prevalence of TOIF.

The erectile function was evaluated by IIEF-15, which includes five domains: erectile function (EF domain; 1-30), orgasmic function (OF domain; 0-10), sexual desire (SD domain; 2-10), intercourse satisfaction (IS domain; 0-15), and overall satisfaction (OS domain; 2-10). The severity of ED was defined based on the EF domain value: severe ED (EF domain < 11), moderate ED (EF domain 11-16), mild to moderate ED (EF domain 17-21), mild ED (EF domain 22-25), and no ED (EF domain 26-30). In addition, the prevalence of depression and anxiety was analyzed by two structured, self-report questionnaires, the Mental Health Inventory-5 (MHI-5) and the Six-Item State-Trait Anxiety Inventory-Short Form (STAI-6), respectively. Depression was primarily considered when the total score of MHI-5 was 52 or lower, and when the total score of STAI-6 equaled to 45 or higher, the patient was diagnosed with anxiety.

All the participants provided at least two semen samples, and the mean values were recorded. Semen analyses were carried out strictly according to the *WHO Laboratory Manual for the Examination and Processing of Human Semen* (fifth edition, 2010).

### 2.3. Statistical Analysis

Patients were divided into two groups based on whether they had ever suffered from TOIF in the last 3 months. Categorical and continuous data were presented as the frequency and mean ± standard deviation, respectively. The chi-squared test was used to compare categorical data between the two groups. The two-tailed Student's *t*-test was used for normally distributed continuous data, and Mann–Whitney's *U* test was used for nonnormally distributed data. Potential factors associated with the occurrence of TOIF were identified by multivariable binary logistic regression analyses. The specific association was evaluated by the adjusted odds ratios (ORs) with their corresponding 95% confidence intervals (CIs). The results were considered significant when two-tailed *p* values < 0.05. The statistical analysis was performed using Stata version 12 (StataCorp LP, College Station, TX).

## 3. Results

### 3.1. Basic Characteristics of the Enrolled Patients

A total of 1128 males who were aware of their female partners' ovulatory time were enrolled, and 264 of them (23.40%) suffered from TOIF. The basic information is listed in Table 1. The mean age was significantly higher in patients with TOIF (*p* < 0.001). Based on the results of the chi-squared test, occurrence of TOIF in males with office work (*p* = 0.011), higher monthly income (*p* < 0.001), smoking (*p* = 0.004), and drinking habits (*p* < 0.001) was significantly higher. In terms of the unique variables of the infertile couples, patients with moderate or worse couple's relationship (*p* = 0.001), lower frequency of sexual intercourse (*p* < 0.001), unknown cause of infertility (*p* = 0.001), lower sperm concentration (*p* < 0.001), and lower progressive motility (*p* < 0.001) were more likely to have TOIF. In terms of the psychological factors, TOIF was associated with anxiety (*p* < 0.001) but not with depression (*p* = 0.391). Besides, the prevalence of hypertension (*p* < 0.001), diabetes (*p* = 0.024), and chronic prostatitis (*p* = 0.001) increased in patients with TOIF.

### 3.2. Factors Associated with TOIF

Possible associated factors for TOIF were further identified by multivariate analysis. The occurrence of TOIF was positively associated with drinking habits (OR: 1.62, 95% CI: 1.14 to 2.29, *p* = 0.007), unknown cause of infertility (OR: 1.65, 95% CI: 1.11 to 2.44, *p* = 0.013), hypertension (OR: 2.35, 95% CI: 1.14 to 4.86, *p* = 0.021), diabetes (OR: 1.53, 95% CI: 1.00 to 2.02, *p* = 0.050), anxiety (OR: 4.51, 95% CI: 2.44 to 8.33, *p* < 0.001), lower frequency of sexual intercourse (OR: 1.82, 95% CI: 1.31 to 2.53, *p* < 0.001), lower sperm concentration (OR: 1.51, 95% CI: 1.03 to 2.21, *p* = 0.035), and erectile dysfunction (OR: 3.89, 95% CI: 2.82 to 5.35, *p* < 0.001). The detailed information is listed in Table 2.

### 3.3. Erectile Function of the Patients with or without TOIF

**Figure 1:**
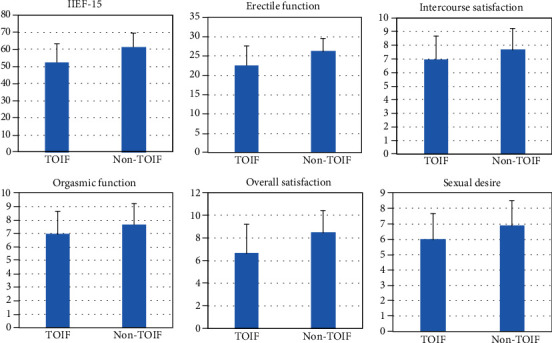
Erectile function of the patients with or without TOIF. ^∗^*p* < 0.001 in all the groups.

The IIEF-15 questionnaire was used to evaluate the erectile function of patients with or without TOIF. Results indicated that the mean EF domain, OF domain, SD domain, IS domain, OS domain, and total score were 22.62 ± 4.96, 6.97 ± 1.70, 6.00 ± 1.67, 10.22 ± 2.38, 6.67 ± 2.57, and 52.48 ± 10.71, respectively, and all of these values were significantly lower than the corresponding indicators in patients without TOIF (*p* < 0.001) (Figure 1). Not only the erectile function but also the subjective feelings including the orgasmic function, sexual desire, intercourse satisfaction, and overall satisfaction were significantly decreased.

**Figure 2:**
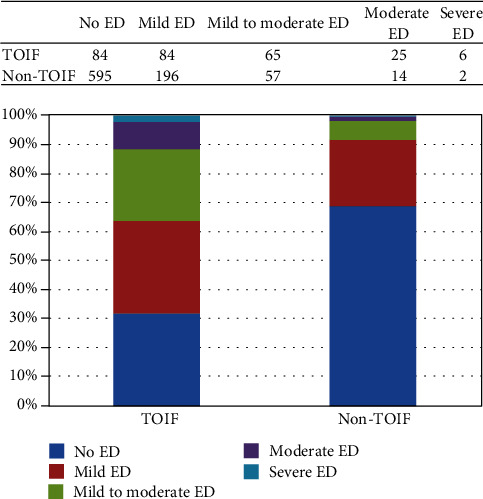
Association between the severity of ED and TOIF. ^∗^*p* < 0.001.

Patients were further grouped into no ED, mild ED, mild to moderate ED, moderate ED, and severe ED to investigate the association between severity of ED and TOIF occurrence. In patients with TOIF, 84 (31.82%) did not have ED, 84 (31.82%) had mild ED, 65 (24.62%) had mild to moderate ED, 25 (9.47%) had moderate ED, and 6 (2.27%) had severe ED. In patients without TOIF, 595 (68.87%) did not have ED, 196 (22.67%) had mild ED, 57 (6.60%) had mild to moderate ED, 14 (1.63%) had moderate ED, and 2 (0.23%) had severe ED. Results demonstrated that the prevalence of TOIF was positively associated with the severity of ED (Figure 2).

## 4. Discussion

Male infertility is a complex problem and affects approximately 7% of the males in modern society. Failure to penetrate the vagina during the sexual partner's ovulatory time can be one common cause for infertility. The concept of TOIF has been put forward by Yang et al. in 2018 [1]. The study which enrolled 4299 patients from 29 urologic clinics in different regions of China reported that 26.2% of the males with timely ovulatory sexual intercourse have TOIF. According to the existing data, TOIF is common and has affected 23.4% of the males of infertile couples. The occurrence is similar to the results of the previous study. Considering the traditional culture of China, males were uppermost in a marital relation and took the responsibility to add a generation to the family tree. The psychological burden of the Chinese young male can be high and subsequently induces a high prevalence of TOIF. Currently, epidemiological data of TOIF in other areas is limited and requires further evaluation.

According to a previous published meta-analysis which investigated the association between lifestyle and sexual function, patients with cigarette smoking and alcohol drinking have 1.41 times and 1.19 times risk of ED, respectively [9]. In the current study, multivariate analyses indicated that smoking and alcohol drinking are also independent associated factors for TOIF. Lifestyle education is important during the treatment of TOIF. Large amounts of clinical studies have demonstrated the association between chronic diseases and ED [10–12]. Patients with cardiovascular diseases or diabetes are more likely to have ED because these conditions share similar associated factors and potential mechanisms [13, 14]. In this cross-sectional study, patients suffering from hypertension and diabetes were more likely to have TOIF. Strengthening the breadth and depth of chronic diseases (such as hypertension and diabetes) management may improve the prognosis of TOIF.

Psychological stress plays an important role in reproductive health and can even affect artificial reproductive technology success [15]. The prevalence of psychological disorders is high in Chinese males of infertile couples, and several studies have reported the negative impact of psychological factors on infertile couples. In 2015, Yang et al. demonstrated that the prevalence of depression and anxiety was 20.8% and 7.8%, respectively, in 771 infertile Chinese males [5]. Compared with patients without psychological disorders, patients with anxiety are more likely to have ED. In the current population, the prevalence of anxiety and depression was similar to this previous study. Notably, the prevalence of anxiety was significantly higher in patients with TOIF. Inability to penetrate the partner's vagina and ejaculation has a direct impact on infertility. When the infertile man experiences TOIF, psychological distress is elevated for both himself and his sexual partner, leading to increased susceptibility to anxiety. In addition to the erectile function and semen analysis results, physicians should strengthen the psychological counseling and humanistic care of the patients.

In terms of the factors associated with infertility, the results indicated that TOIF was positively associated with unknown cause of infertility and lower sperm concentration. The sperm concentration can directly reflect the severity of male infertility. The modern assisted reproductive technique (ART) allows patients with severe oligozoospermia to have their own child by using just tiny amounts of sperm [16, 17]. However, the management of patients with azoospermia is still challenging and the reproductive outcomes are usually unsatisfied. In addition, the fear of unknown causes of infertility can also increase the psychological stress of these patients. These factors may affect the erectile function and subsequently result in TOIF. In addition, these factors of infertility are firmly associated with testicular dysgenesis syndrome (TDS). TDS is also a risk factor of ED or worsened ED and may subsequently result in TOIF.

ED is common in males with infertility. The prevalence of ED has been reported by several studies, ranging from 18% to 57% [18, 19]. The association between ED and TOIF has also been identified. Similar to the results of Dr. Yang et al. [1], ED is an independent associated factor for TOIF in the current study. To further investigate the subjective satisfaction of the patients with TOIF, the IIEF-15 questionnaire was used. The results indicated that the total IIEF-5 scores, erectile function, orgasmic function, sexual desire, intercourse satisfaction, and overall satisfaction were significantly decreased in patients with TOIF. Not only the erectile function but also the subjective feelings of the patients should be focused on. Notably, severe ED just occupied a small proportion in patients with male infertility, ranging from 2.4 to 2.9% [20, 21]. In the current population, severe ED accounts for 2.27% of the patients with TOIF. Most of these patients suffered from mild or mild to moderate ED. The severity of ED is positively associated with TOIF occurrence. While clinicians should focus on the overall satisfaction of the patients, treatment of ED is also very important, especially in those with severe ED.

There were several strengths of the current study. First, a relatively large number of patients were enrolled which enabled further multivariate analyses. Second, semen analyses and psychological status were analyzed in all the participants. Despite the aforementioned advantages, sexual hormones were not detected and this was a limitation. Concerning the important role of hormones, including sexual hormones, thyroxine, and melatonin, in erectile function, it is necessary for future studies researching on this field.

## 5. Conclusions

TOIF is associated with lower semen concentration, anxiety, and other comorbidities including hypertension and diabetes. Clinicians are required to focus on these associated factors in addition to improve the erectile function of the males of infertile couples.

## Figures and Tables

**Table 1:** Basic characteristics of the patients with or without TOIF.

Parameters	Subjects		*p* value
	TOIF (*n* = 264)	Non-TOIF (*n* = 864)	
Age (y), mean ± SD	34.39 ± 6.19	32.18 ± 5.25	<0.001
BMI (kg/m^2^), mean ± SD	24.71 ± 3.13	24.81 ± 3.18	0.595
Residence			0.118
Rural	32 (12.12)	141 (16.32)	
Urban	232 (87.88)	723 (83.68)	
Education level, *n* (%)			0.996
No higher than junior high school	24 (9.09)	77 (8.91)	
High school	47 (17.80)	154 (17.82)	
University and above	193 (73.11)	633 (73.27)	
Monthly income (RMB), *n* (%)			<0.001
<5000	78 (29.55)	403 (46.64)	
5001-10000	116 (43.94)	310 (35.88)	
10001-20000	46 (17.42)	103 (11.92)	
>20000	24 (9.09)	48 (5.56)	
Occupation, *n* (%)			0.011
Office work	141 (53.41)	398 (46.07)	
Manual labor work	71 (26.89)	216 (25.00)	
Other	52 (19.70)	250 (28.93)	
Smoking, *n* (%)			0.004
Yes	106 (40.15)	267 (30.90)	
No	158 (59.85)	597 (69.10)	
Alcohol consumption, *n* (%)			<0.001
Yes	112 (42.42)	239 (27.66)	
No	152 (57.58)	625 (72.34)	
Couple's relationship, *n* (%)			0.001
Good	238 (90.15)	828 (95.83)	
Moderate or worse	26 (9.85)	36 (4.17)	
Marital status, *n* (%)			0.292
Primary	250 (94.70)	827 (95.72)	
Secondary	14 (5.30)	37 (4.28)	
Frequency of intercourse (*n*/month), *n* (%)			<0.001
<5	126 (47.73)	235 (27.20)	
5-8	113 (42.80)	416 (48.15)	
>9	25 (9.47)	213 (24.65)	
Medical history			
Type of infertility, *n* (%)			0.182
Primary	182 (68.94)	633 (73.26)	
Secondary	82 (31.06)	231 (26.74)	
Duration of infertility, *n* (%)			0.101
<2	70 (26.52)	205 (23.73)	
2-4	112 (42.42)	431 (49.88)	
>4	82 (31.06)	228 (26.39)	
Cause of infertility, *n* (%)			0.001
Known	192 (72.73)	709 (82.06)	
Unknown	72 (27.27)	155 (17.94)	
Hypertension, *n* (%)			<0.001
Yes	24 (9.09)	22 (2.55)	
No	240 (90.91)	842 (97.45)	
Diabetes, *n* (%)			0.024
Yes	9 (3.41)	10 (1.16)	
No	255 (96.59)	854 (98.84)	
Chronic prostatitis, *n* (%)			0.001
Yes	47 (17.80)	90 (10.42)	
No	217 (82.20)	774 (89.58)	
Varicocele, *n* (%)			0.060
Yes	72 (27.27)	291 (33.68)	
No	192 (72.73)	573 (66.32)	
Depression, *n* (%)			0.391
Yes	38 (14.39)	145 (16.78)	
No	226 (85.61)	719 (83.22)	
Anxiety, *n* (%)			<0.001
Yes	38 (14.39)	34 (3.94)	
No	226 (85.61)	830 (96.06)	
Semen quality, *n* (%)			
Sperm concentration (10^6^/mL), *n* (%)			<0.001
<5	38 (14.39)	32 (3.70)	
5-15	64 (24.24)	247 (28.59)	
>15	162 (61.37)	585 (67.71)	
Progressive motility (%), *n* (%)			<0.001
<5	17 (6.44)	14 (1.62)	
5-20	37 (14.01)	67 (7.76)	
20-32	102 (38.64)	381 (44.10)	
>32	108 (40.91)	402 (46.52)	

**Table 2:** Multivariate analysis of the association between study variables and TOIF occurrence.

Parameters	Adjusted OR	95% CI	*p* value
Age > 32 years	1.27	0.90 to 1.79	0.177
Smoking	1.42	1.00 to 2.02	0.052
Drinking	1.62	1.14 to 2.29	0.007
Monthly income > 10000 RMB	1.37	0.92 to 2.04	0.124
Office work	1.19	0.86 to 1.64	0.290
Secondary infertility	0.97	0.67 to 1.40	0.875
Unknown cause of infertility	1.65	1.11 to 2.44	0.013
Hypertension	2.35	1.14 to 4.86	0.021
Diabetes	1.53	1.00 to 2.02	0.050
Chronic prostatitis	1.38	0.89 to 2.14	0.152
Varicocele	1.05	0.83 to 1.32	0.664
Anxiety	4.51	2.44 to 8.33	<0.001
Depression	0.73	0.48 to 1.12	0.149
Frequency of intercourse < 5/month	1.82	1.31 to 2.53	<0.001
Sperm concentration < 15∗10^6^/mL	1.51	1.03 to 2.21	0.035
Progressive motility < 32%	1.17	0.82 to 1.68	0.392
IIEF‐EF < 26	3.89	2.82 to 5.35	<0.001

## Data Availability

The data supporting the conclusions of this article is included within the article.

## Abbreviations

TOIF: Timely ovulatory intercourse failure
ED: Erectile dysfunction
MHI-5: Mental Health Inventory-5
STAO-6: Six-Item State-Trait Anxiety Inventory-Short Form
IIEF-15: International Index of Erectile Function 15.
